# Central-line associated bacteremia in intensive care units in Uruguay. 2007-2010 national data

**DOI:** 10.1186/1753-6561-5-S6-O9

**Published:** 2011-06-29

**Authors:** SE Guerra, H Albornoz, R Rosa, G Rios, H Bagnulo, T Camou, A Galiana, A Pilatti, G Reherman, M Godino

**Affiliations:** 1 Infection Control Committee, Ministry of Public Health, Montevideo, Uruguay

## Introduction / objectives

Intensive Care Unit (ICUs) patients account for a large proportion of hospital infections (HI) and Central Line Associated Bloodstream Infections (CLAB) are one of the most common. We determined the incidence of CLAB in adult (A) and neonatal (N) intensive care units and evaluated the national response to bundle interventions.

## Methods

A national surveillance HI system was implemented since 2006 and prospective surveillance is performed using NNISS criteria. The system is mandatory, data are recorded online and sent to the Ministry of Health. Data are audited annually, results are published each year and recommendations were made. Interventions were: alcohol hand rub, alcoholic chlorhexidine for skin antisepsis and maximal barrier precautions for catheter insertion.

## Results

Neonatal and adult ICUs from 53 hospitals reported. CLAB rate in NICU was 7.9/1000 catheter-days (474 episodes in 60147 catheter-days), annual incidence lowered in all strata of weight between 2007 and 2010 (15.8 to 8.6, 11 to 6, 6.7 to 1.7 and 4 to 2.3/1000 catheter/days, for < 1000, 1001-1500, 1501-2500 and >2500 gr, respectively). CLAB rate in adult ICUs was 2.7/1000 catheter-days (666 episodes in 247.272 catheter-days), annual incidence was 3.8, 2.6, 2.7 and 1.9/1000 catheter/days, for 2007, 2008, 2009 and 2010, respectively. The main microorganisms were S. coagulase negative (AICU 17.3%, NICU 30.6%), *S. aureus* (AICU 14.7%, NICU 14%), *K. pneumoniae* (AICU 12%, NICU 7.1%), *Ps. aeruginosa* (AICU 6.3%, NICU 5.4%) and Candida sp (AICU 3.9%, NICU 6.8%).

## Conclusion

CLAB surveillance, data publishing and national recommendations contributed to lower the national rates in adults and neonatal ICUs in Uruguay.

## Disclosure of interest

None declared.

